# 

**Published:** 1976

**Authors:** 


The
Bristol Medico-Chirurgical Journal
(Incorporating the Medical Journal of the South-West)
Established 1883

Vol. 91 (iii/iv) JULY/OCTOBER 1976 No. 339/340

### BRISTOL MEDICO-CHIRURGICAL JOURNAL

EDITOR: D. S. REEVES, M.B., M.R.C.Path.

Published Quarterly

Annual Subscription £4 Post Free

### BRISTOL MEDICO-CHIRURGICAL SOCIETY

**President 1976/77:** Dr. S. J. Silvey.

**President-elect:** Dr. A. T. M. Roberts.

**Honorary Secretary:** A. J. Webb, Esq., 7 Percival Road, Bristol 8.

**Honorary Treasurer:** Dr. J. B. Penry, Southmead, Hospital, Bristol.

The Society was founded in 1874. In 1883 publication of the Journal began and has continued quarterly ever since then. During the years 1949 to 1962 it appeared under the title of "The Medical Journal of the South West".

### THE BRISTOL MEDICO-CHIRURGICAL JOURNAL

**Editorial Committee**

Editor Dr. D. S. Reeves, Southmead Hospital, Bristol.

Assistant Editor Dr. A. P. Radford

Members Dr. Rhys Davies.
Dr. M. B. Lennard.
Dr. D. W. Wright.
The Hon. Treasurer and Hon. Secretary of the Society.

Secretary Mr. B. P. Jones, The Library, Medical School, University Walk, Bristol BS8 1TD.

### NOTICE TO CONTRIBUTORS

The Journal is especially concerned to provide a place for the recording of medical thought, interests, and practice in and around Bristol. It therefore welcomes articles that, as well as being of immediate interest to members, will document the local and contemporary medical scene for our successors.

Original articles are invited provided that they have not been published elsewhere. References in the text should be by author's name, with year of publication in parenthesis; they should be listed at the end in alphabetical order, the name of each journal or book being given in full—not abbreviated—and the title of the article added. Illustrations should be supplied ready for reproduction. Line drawings should be in Indian ink on firm white paper or board. The author will be informed if it is found necessary to ask him to pay for the making of blocks.

The following contributions will be especially welcome; notes of forthcoming events, meetings held, degrees conferred, staff appointments, honours and public appointments and other local news.

### University of Bristol

### FINAL EXAMINATIONS FOR THE DEGREES OF MB, ChB

### June 1976

### Pass

**WITH HONOURS**

Rebecca Barton DUNN — with Distinctions in Child Health and in Pathology
Ian Arthur EYRE-BROOK — with Distinction in Surgery
Catherin Mary GILBERT — with Distinction in Pathology
Peter James HARNEY — with Distinction in Community Medicine
Charles William HUTTON — with Distinction in Mental Health
Corinne Alison ILLINGWORTH — with Distinctions in Surgery, Child Health and in Obstetrics and Gynaecology.
Carole Mary JUNEMAN — with Distinctions in Obstetrics and Gynaecology
Mary Elizabeth McGRAW — with Distinction in Child Health
Charles Richard SHAWCROSS — with Distinction in Mental Health

**PASS**

Graeme James Mackay ALEXANDER
Jennifer Elisabeth AMERY
Trevor George ARMITAGE
Valerie Jane AUSTIN
Albert Anthony BARCELLOS
Ian Frederick BELL
Philip BOSWORTH — with Distinction in Community Medicine
Phillip Anthony BRIGHT
Duncan Frederick Strathdee BURWOOD
Sheila Anne CALLOW
Graeme Paul CALVER
Julie Mildred CAMSEY
Michael CARPENTER
Martin James CARROLL
Jonathan CHAPPELL
Michael Anthony CLARKE
Ceridwen Elizabeth COLE
Rosalyn Mary COLEMAN
Graeme COOKSEY
Catherine COULSON
Robert James CRAWFORD
Jack Maciej CZAUDERNA
Tawfique Khan DANESHMEND — with Distinction in Pathology
Janie Christine DARLING
Sunil Jayantilal Sunderlal DESAI
Rosemary Anne DIDCOCK
Gordon Neale DUTTON
Catherine Anne ELLIOT
John Charles FELTON
Royston Edward FEY
Robin Brian Dansey FISHER
Christopher Charles GAFFNEY
Clare Elizabeth GILBERT
Christopher GILBERTHORPE
Annabelle Frances GLASIER
David Henry GLOVER
Robert Hugh GODDARD
Peter John GRANT
Stephen William GREEN
Roger Newton GREENWOOD
Nicholas Duncan GROVES
Ian Samuel HAMILTON
Anthony Andrew HANCOCK
Gaye Victoria HARDIMAN
Barbara Anna HARNEY
George Gordon HARTNELL
Lauritta Karen Sonia HAWKINS — with Distinction in Obstetrics and Gynaecology
Joanne Mary HEAF
Andrew John HENLEY
Rosalyn Jill HEYWOOD
Robert Charles HIAM
Anne Grafton HICKS
David Colin Wood HILTON
Anthony George HOLBROOK
Colin Geoffrey HOLLIDAY
Anne HONG
Catherine Mary HOWARD
Sezgin Mehmet ISMAIL
Anthony Nicholas JONES
Guinevere Ann JONES
Janice Elizabeth JONES
John David KNEESHAW
Nicholas George LAVIES
David Neal LONGDON
Paul George MATTISON
Charlotte Veronica MELIA
Michael Ronald MILLAR
Keith MYERS
Lesley Margaret NEAL
Geoffrey Hannel NEWMAN
Maureen O'LEARY
Iain Henry St. George OSWALD
Harriett Maude Jennie PLATTEN
Elizabeth Ann PROCTER
John Andrew ROBERTS
David Hugh RYAN
Patricia SCOTT
Stewart William SCOTT

Andrew Charles SEAMAN
Richard James SHERRIFF
Phyllis Ruth SIMPSON
Michael Edward SINCLAIR
Neil Cranson SONI
Gillian Margaret STOWELL
Julie STRAWFORD
Michael Andrew SWIFT
Nigel John THEAKER
Josephine Ann TITCOMBE
Kenneth James TREMAIN
Paul Brian VEALE
Michael Stephen Carroll WEBB
Simon Paul WERNICK
Richard Lloyd WHARTON
Sally Ann WILKINSON
John Frederick WILSON
Frances Ann WOOD
Andrew John WRIGHT
Hilary Mary WYATT
William Nicholas WYKES
Ian Charles WYNNE
David John YARNALL
Judith Margaret YELTON

### A SELECTION OF RECENT ADDITIONS TO THE MEDICAL LIBRARY

ALEXANDER, J. O.: Dermatitis herpetiformis (Saunders).

BAER, G. M.: The natural history of rabies. 2 vols. (Acad Pr.).

BAKER, A. A.: Comprehensive psychiatric care (Blackwell).

BALLS, M. and MONNICKENDAM, M. A.: Organ culture in biomedical research (Cambridge Univ. Pr.).

BENNETT, A. E.: Communication between doctors and patients (Oxford Univ. Pr.).

BESSIS, M. and BRECHER, G.: Unclassifiable leukemias (Springer).

BIRCH, G. C. and PARKER, K. J.: Vitamin C (Applied Science Publishers).

BLANDY, J.: Urology. 2 vols. (Blackwell).

BRADFORD, L. J.: Physiological measures of the audiovestibular system( Acad. Pr.).

BRONSTEIN, E. S.: Laparoscopy for sterilization (Lloyd-Luke).

BURKHOLDER, P. M.: Atlas of human glomerular pathology (Harper & Row).

BURKITT, D. P. and TROWELL, H. C.: Refined carbohydrate foods and disease (Acad. Pr.)

CALNAN, J.: One way to do research (Heinemann).

CAMERON, J. M. and RAE, L. J.: Atlas of the battered child syndrome (Churchill Livingstone).

CAMERINI-DAVALOS, R. A. and COLE, H. S.: Early diabetes in early life (Acad. Pr.)

CANIZARES, O.: Clinical tropical dermatology (Blackwell).

CANTWELL, D. P.: The hyperactive child (Spectrum).

CARR, J.: Young children with Down's syndrome (Butterworth).

CHALMERS, J. A.: Andometriosis (Butterworth).

CLIFF, W. J.: Blood vessels (Cambridge Univ. Pr.)

CLINE, M. J.: The white cell (Harvard Univ. Pr.)

COGAN, D. G.: Ophthalmic manifestations of systemic vascular disease (Saunders).

CUNLIFFE, W. J. and COTTERILL, J. A.: The acnes (Saunders).

CURTIS, J. R. and WILLIAMS, G. B.: Clinical management of chronic renal failure (Blackwell).

DAVIDSON, J. K.: Aseptic necrosis of bone (Excerpta Medica).

DAVIES, D. S. and REID, J. L.: Central actions of drugs in blood pressure regulation (Pitman).

DAVISON, A. N.: Biochemistry and neurological disease (Blackwell).

DE BOER, J. et al: An introduction to experimental surgery (Excerpta Medica).

DECKER, K. and BACKMUND, H.: Paediatric neuroradiology (Butterworth).

DEMLING, L. et al: Atlas of enteroscopy (Springer).

DENDY, P. P.: Human tumours in short term culture (Acad. Pr.)

DISAIA, P. J. et al: Synopsis of gynecologic oncology (Wiley).

DUMONDE, D. C.: Infection and immunology in the rheumatic diseases (Blackwell).

DYCK, P. J. et al: Peripheral neuropathy (Saunders).

EDIS, A. J. et al: Manual of endocrine surgery (Springer).

EDWARDS, W. H.: Vascular surgery (University Park Pr.).

EHRLICH, G. E.: Oculocutaneous manifestations of rheumatic diseases (Karger).

EISENBACH, G. M. et al: Proteinuria (Karger).

FASSBENDER, H. G.: Pathology of rheumatic diseases (Springer).

FISCHER, J. E.: Total parenteral nutrition (Little, Brown).

FORREST, A. and AFFLECK, J. W.: New perspectives in schizophrenia (Churchill Livingstone).

FOX, H. and LANGLEY, F. A.: Tumours of the ovary (Heinemann).

FRAUMENI, J. E.: Persons at high risk of cancer (Acad. Pr.).

GIROUD, J. P. et al: Future trends in inflammation, 2 (Birkhauser V1g).

GLENN, J. F.: Urologic surgery. Ed. 2 (Harper & Row).

GLENN, T. M.: Steroids and shock (University Park Pr.).

GRABB, W. C. and MYERS, M. B.: Skin flaps (Little Brown).

GRAMIAK, R. and WAAG, R. C.: Cardiac ultrasound (Mosby).

GREENSPAN, E. M. ed: Clinical cancer chemotherapy (Raven Pr.)

GRUNEBAUM, H. et al: Mentally ill mothers and their children (Chicago Univ. Pr.).

HALPERN, S. L. et al: Nutrition and metabolism in medical practice (Futura).

HARPER, R.: The new psycotherapies (Prentice-Hall).

HARRISON, T. S. et al: Surgical disorders of the adrenal gland (Grune & Stratton).

HEMMINGS, W. A.: Maternofaetal transmission of immunoglobulins (Cambridge Univ. Pr.).

HERXHEIMER, H. A.: A guide to bronchial asthma (Acad. Pr.).

HIRSCH, S. R. and LEFF, J. P.: Abnormalities in parents of schizophrenics (Oxford Univ. Pr.).

HODKINSON, H. M.: An outline of geriatrics (Acad. Pr.).

HOPE-STONE, H. F.: Radiotherapy in modern clinical practice (Crosby Lockwood Staples).

HOWELLS, J. G.: Modern perspectives in the psychiatry of old age (Churchill Livingstone).

IONESCU, M. and WOLLER, G. H.: Current techniques in extra-corporeal circulation (Butterworth).

ISACCSON, R. L. and PRIBRAM, K. H.: The hippocampus. 2 vols (Plenum Pr.).

JOHNSTON, G. S. and JONES, E.: Breast cancer diagnosis (Plenum Pr.).

JONES, I. C. and HENDERSON, I. W.: General comparative and clinical endocrinology of the adrenal cortex. Vol. 1 (Acad. Pr.).

JONES, P. G. and CAMPBELL, P. E.: Tumours of infancy and childhood (Blackwell).

JOSS, E. E.: Growth hormone deficiency in childhood (Karger).

KAPLAN, H. S.: The new sex therapy (Balliere).

KINCAID-SMITH, P.: The kidney: a clinico-pathological study (Blackwell).

KISSANE, J. M.: Pathology of infancy and childhood. Ed. 2 (Mosby).

KLAINER, A. S. and GEIS, I.: Agents of bacterial disease (Harper & Row).

KOHN, R. and WHITE, K. L.: Health care: an international study (Oxford Univ. Pr.).

LAIDLAW, J. and RICHENS, A.: A textbook of epilepsy (Churchill Livingstone).

LENNERSTAND, G. and BACH-Y-RITA, P.: Basic mechanisms of ocular motility and their clinical implications (Pergammon Pr.).

LEOPOLD, G. R. and ASHER, W. M.: Fundamentals of abdominal and pelvic ultrasonography (Saunders).

LOWBURY, E. J. L. et al: Control of hospital infection (Chapman & Hall).

LYNCH, H. T.: Cancer genetics (Thomas).

MACGILLIVRAY, I. et al: Human multiple reproduction (Saunders).

MATHEWS, M. B.: Connective tissue (Springer).

MAYOR, G. and ZINGG, E. J: Urologic surgery (Thieme).

McDICKEN, N.: Diagnostic ultrasonics (Crosby Lockwood Staples).

McCANCE, R. A. and WIDDOWSON, E. M.: Calorific deficiencies and protein deficiencies (Churchill Livingstone).

MEITES, J. et al: Pioneers in neuroendocrinology (Plenum Pr.).

MIER, P. D. and COTTON, D. W. K.: The Molecular biology of skin (Blackwell).

MILUNSKY, A.: Prevention of mental retardation and genetic disease (Saunders).

MOBIN-UDDIN, K.: Pulmonary thromboembolism (Thomas).

MONIF, G. R. G.: Infectious diseases in obstetrics and gynecology (Harper & Row).

MORGAN, W. K. C. and SEATON, A. Occupational lung diseases (Saunders).

MORRISON, A. W.: Management of sensorineural deafness (Butterworth).

NAJARIAN, J. S. and DELANEY, J. P.: Surgery of the gastrointestinal tract (Symposia Specialists).

NAVARATNAM, V.: The human heart and circulation (Acad. Pr.).

ODASHIMA, S. et al: Recent topics in chemical carcinogenesis (University Park Pr.)

OSOFSKY, H. J. and OSOFSKY, J. D.: The abortion experience: psychological and medical impact (Harper & Row).

PARSONS, J. A.: Peptide hormones (Macmillan).

PAYNE, J. P. and HILL, D. W.: Oxygen measurements in biology and medicine (Butterworth).

POPPER, H. and BECKER, K.: Collagen metabolism in the liver (Stratton).

PORTMANN, M. et al: The internal auditory meatus: anatomy, pathology and surgery (Churchill Livingstone).

PUGH, R. C. B.: Pathology of the testis (Blackwell).

RAHI, A. H. S.: Immunopathology of the eye (Blackwell).

RAMOT, B. et al: Genetic polymorphisms and diseases in man (Acad. Pr.).

ROSS, P. and DU BOULAY, G. H.: An atlas of normal vertabral angiograms (Butterworth).

RUBIN, M. I. and BARRATT, T. M.: Pediatric nephrology (Williams & Wilkins).

SASAHARA, A. A. et al: Pulmonary emboli (Grune & Stratton).

SCHILLER, K. F. R. and SALMON, P. R.: Modern topics in gastrointestinal endoscopy (Heinemann).

SCHIRIRE, T. Surgical emergencies: diagnosis and management (Heinemann).

SCHOTTENFELD, D.: Cancer epidemiology and prevention: current concepts (Thomas).

SCHUKNECHT, H. E.: Pathology of the ear (Harvard Univ. Pr.).

SHAFRIR, E.: Contemporary topics in the study of diabetics and metabolic endocrinology (Acad. Pr.).

SILBER, E. N. and KATZ, L. N.: Heart disease (Balliere).

SMITH, S. M.: The battered child syndrome (Butterworth).

SPRANGER, J. W. et al: Bone dysplasias (Saunders).

STOLL, B. A.: Risk factors in breast cancer (Heinemann).

STUART, A. E. et al: Applied surgical pathology (Blackwell).

SYMINGTON, T. and CARTER, R. L.: Scientific foundations of oncology (Heinemann).

TIXIER-VIDAL, A. and FARQUHAR, M. G.: The anterior pituitary (Acad. Pr.).

VALLANCE-OWEN, J.: Diabetes: its physiological and biochemical basis (University Park Pr.).

VIANNA, N. J.: Lmyphoreticular malignancies (M.T.P.).

WALKER, F. C.: Modern stoma care (Churchill Livingstone).

WEISSMANN, G.: Mediators of inflammation (Plenum Pr.).

WICKRAMASINGHE, M. B.: Human bone marrow (Blackwell).

YANOFF, M. and FINE, B. S.: Ocular pathology: text and atlas (Harper & Row).

YAP, P. M.: Comparative psychiatry: a theoretical framework (Toronto Univ. Pr.).

ZUPINGER, K. A.: Hypoglycemia in childhood (Karger).

